# No more Mr Nice Guy – implementation of mandatory seasonal influenza immunization for all personnel

**DOI:** 10.1186/1753-6561-5-S6-P276

**Published:** 2011-06-29

**Authors:** JP Parada, M Koller, J Carlson-Steinmetz, B Gaughan, C Schleffendorf, M Capelli-Schellpfeffer, P Hindle

**Affiliations:** 1 Medicine, Loyola University, Maywood, IL, USA; 2 Nursing, Loyola University, Maywood, IL, USA

## Introduction / objectives

In the US, immunization for many vaccine preventable illnesses are mandated as a condition of employment, but Influenza (flu) has not been mandatory. Despite 1986 Center for Disease Control recommendations that healthcare workers be vaccinated annually for flu, the US national average is 61.9%. Patient & employee safety / preventing nosocomial infections are central to quality care. So, we sought to achieve universal flu immunization for all personnel.

## Methods

With firm commitment of leadership, seasonal flu immunization was mandated as condition of employment. A multidisciplinary task force guided interventions/policy changes. We launched an internal media blitz. Paper & electronic tracking systems measured compliance. We developed a formal exemption process. Nursing administration & pharmacy coordinated vaccination.

## Results

Pre-mandatory immunization, our baseline 3-year flu vaccination rate was 65% (range 62-72%). In the 2 years since mandatory flu immunization was instituted we achieved a > 99% immunization rate. In 2009, 7795/7854 (99.2%) personnel received vaccine, 52 (0.7%) were exempted for religious/medical reasons, and 9 employees (0.1%) refused vaccination, choosing to terminate employment. In 2010, 8030/8091 (99.2%) were vaccinated, 60 (0.7%) were exempted, and 1 person (0.01%) chose to terminate employment.

## Conclusion

100% participation in flu immunization in the healthcare setting is possible and near universal flu immunization is achievable and sustainable. It is doubtful that these levels of immunization would be achievable if not as a condition of employment. We believe that patient/employee safety have been enhanced, and quality of care is improved.

## Disclosure of interest

J. Parada Grant/Research support from Astellas, Roche, Speaker's Bureau of Optimer, Consultant for Merck, M. Koller: None Declared, J. Carlson-Steinmetz: None Declared, B. Gaughan: None declared, C. Schleffendorf: None declared, M. Capelli-Schellpfeffer: None declared, P. Hindle Consultant for Medline.

